# Oral Antibiotics for Bacteremia and Infective Endocarditis: Current Evidence and Future Perspectives

**DOI:** 10.3390/microorganisms11123004

**Published:** 2023-12-18

**Authors:** Gerasimos Eleftheriotis, Markos Marangos, Maria Lagadinou, Sanjay Bhagani, Stelios F. Assimakopoulos

**Affiliations:** 1 Division of Infectious Diseases, Department of Internal Medicine, Medical School, University of Patras, University Hospital of Patras, Rion, 26504 Patras, Greece; makiseleftheriotis@yahoo.gr (G.E.); marangos@upatras.gr (M.M.); mlagad@upatras.gr (M.L.); 2 Department of Infectious Diseases and HIV Medicine, Royal Free London NHS Foundation Trust, London NW3 2QG, UK; s.bhagani@ucl.ac.uk

**Keywords:** bacteremia, endocarditis, oral treatment, *Enterobacterales*, *Staphylococcus*, *Streptococcus*, *Enterococcus*, *Pseudomonas*

## Abstract

Bacteremia and endocarditis are two clinical syndromes that, for decades, were managed exclusively with parenteral antimicrobials, irrespective of a given patient’s clinical condition, causative pathogen, or its antibiotic susceptibility profile. This clinical approach, however, was based on low-quality data and outdated expert opinions. When a patient’s condition has improved, gastrointestinal absorption is not compromised, and an oral antibiotic regimen reaching adequate serum concentrations is available, a switch to oral antibacterials can be applied. Although available evidence has reduced the timing of the oral switch in bacteremia to three days/until clinical improvement, there are only scarce data regarding less than 10-day intravenous antibiotic therapy in endocarditis. Many standard or studied oral antimicrobial dosages are smaller than the approved doses for parenteral administration, which is a risk factor for treatment failure; in addition, the gastrointestinal barrier may affect drug bioavailability, especially when the causative pathogen has a minimum inhibitory concentration that is close to the susceptibility breakpoint. A considerable number of patients infected by such near-breakpoint strains may not be potential candidates for oral step-down therapy to non-highly bioavailable antibiotics like beta-lactams; different breakpoints should be determined for this setting. This review will focus on summarizing findings about pathogen-specific tailoring of oral step-down therapy for bacteremia and endocarditis, but will also present laboratory and clinical data about antibiotics such as beta-lactams, linezolid, and fosfomycin that should be studied more in order to elucidate their role and optimal dosage in this context.

## 1. Introduction

Bacteremia and infective endocarditis are two clinical entities associated with increased morbidity and mortality. Most of the time, hospital admission is necessary, leading to increased hospital stays and healthcare costs. To date, there are no guidelines for oral step-down therapy in patients with bacteremia when the patient’s clinical condition has improved. As a result, this topic is still a matter of debate, and lots of physicians are reluctant to implement it in their everyday practice, especially among patients with *S. aureus* bacteremia or infective endocarditis [1]. A previous survey has showed that there are geographical discrepancies regarding treatment strategies between infectious disease specialists, and lots of healthcare providers apply oral step-down only for some sources of bacteremia (more commonly acute bacterial skin/skin structure infections (ABSSSI) and urinary sources) [2].

This strategy, except for cost minimization, could reduce healthcare-associated complications, including infections, and discomfort among patients who would otherwise receive inpatient or outpatient parenteral antimicrobial therapy (OPAT) [3,4]. OPAT is not adverse-event-free: 9% of 1461 OPAT courses from a study conducted in the US had at least one intravenous (IV) access complication requiring intervention [5]. A significant event in 14.5% of the cases was reported in another study [6]. The complication rate rises when a long-term IV antibiotic regimen is opted for. The mean time for the first complication reported in a prospective cohort for peripherally inserted central catheters (PICCs) was 16.1 days, whereas the overall complication rate was 30.2% including occlusion, accidental removal, infection, and thrombosis [7]. Oral therapy also improves patients’ quality of life by reducing in-hospital length of stay, which is one of the axes of antimicrobial stewardship, and eliminates restriction in daily activities and social life that a central catheter can cause, as well as other difficulties such as mobility restriction due to the IV catheter [6,8]. As a result, patients likely prefer oral antibiotic therapy [9].

In recent years, several studies, the majority of them retrospective, have been published or are ongoing in this setting, mostly using oral antibiotic monotherapy for bacteremia and combination therapy for endocarditis, even though it is still unclear if antibiotic combination is indeed superior in some cases or if it simply serves as a safety net for patients who will have subtherapeutic blood levels of the first-choice antibiotic.

## 2. Methods

A systematic literature search was conducted using PubMed and PubMed Central from inception until 15 October 2023. Several research terms were used to identify relevant literature: “oral” AND “bacteremia”, “oral” AND “endocarditis”, “oral penicillin” OR “oral amoxicillin” OR “oral amoxicillin/clavulanate” OR “oral cloxaxillin” OR “oral dicloxacillin” OR “oral cefalexin” OR “cefuroxime axetil” OR “cefaclor” OR “cefprozil” OR “cefixime” OR “cefditoren” OR “cefpodoxime” OR “ciprofloxacin” OR “levofloxacin” OR “moxifloxacin” OR “oral fluoroquinolones” OR “trimethoprim-sulfamethoxazole” OR “clindamycin” OR “linezolid” OR “tedizolid” OR “rifampin” OR “rifampicin” OR “metronidazole” OR “oral fosfomycin” AND “bacteremia”, “oral penicillin” OR “oral amoxicillin” OR “oral amoxicillin/clavulanate” OR “oral cloxaxillin” OR “oral dicloxacillin” OR “oral cefalexin” OR “cefuroxime axetil” OR “cefaclor” OR “cefprozil” OR “cefixime” OR “cefditoren” OR “cefpodoxime” OR “ciprofloxacin” OR “levofloxacin” OR “moxifloxacin” OR “oral fluoroquinolones” OR “trimethoprim-sulfamethoxazole” OR “clindamycin” OR “linezolid” OR “tedizolid” OR “rifampin” OR “rifampicin” OR “metronidazole” OR “oral fosfomycin” AND “endocarditis”. Results were screened for appropriateness by the first and the last author, according to title and abstract. Most relevant papers were further assessed by full content; their references were also reviewed and assessed when appropriate. Only articles published in the English language were included. Article types included clinical studies, experimental studies, clinical trials, and reviews.

## 3. Effect of Acute Febrile Infection on Oral Antibiotic Absorption

Fever and acute infection in non-critically ill patients do not seem to affect oral absorption of antibiotics when the infection is outside the gastrointestinal tract [10]. In addition, a retrospective study from two Brazilian intensive care units showed no difference in mortality when in-hospital step-down to oral antimicrobials was applied in initially septic patients (most often from pneumonia) that had reached clinical stability [11]. A proportion 60.9% of the cases received fluoroquinolones, which seems rational because oral bioavailability of levofloxacin is similar to IV, even in critically ill patients [11,12].

### 3.1. Bacteremia and Oral Antibiotics

#### 3.1.1. Gram-Negative Bacteremia

##### *Enterobacterales* Bacteremia: Clinical Efficacy Data and Upcoming Trials

Plenty of evidence regarding oral step-down in cases of bacteremia caused by *Enterobacterales* is available from retrospective studies. All treatment outcomes have been analyzed in several publications compared with patients who received parenteral antibiotics for the whole course of infection (re-initiation of IV antibiotics, microbiological recurrence, bacteremia-related death within 21 days of negative blood cultures, 30-day urinary tract infection (UTI) recurrence, and 30-day and 90-day bacteremia recurrence and mortality); no statistically significant difference was identified in any of them [13,14,15,16,17]. These differences remained non-significant between different sources of bacteremia (UTI, gastrointestinal tract, catheter-related bloodstream infection (CRBSI), and primary bacteremia) [13,14,15,16,18,19]. Oral de-escalation with fluoroquinolones has been also reported even in patients with hematological or solid malignancies without prolonged bacteremia or neutropenia for >5 days [20]. Appropriate source control upon indications (e.g., biliary drainage, removal of infected catheter, resolution of urinary obstruction) seems to be a prerequisite before oral switch [14,21,22,23]. In addition, some studies have provided us evidence about significantly higher rate of IV-line complications in those receiving IV-only antibiotics [13,20]. Patients who received only parenteral regimens stayed in the hospital for a median of 6–7 days, whereas the group which underwent oral de-escalation had a shorter median length of stay (4–5 days) [13,14,15].

Small, randomized trials have been published in this setting, too. Park et al. revealed no difference in bacterial eradication, infection recurrence or 30-day mortality studying patients with bacteremic cholangitis after successful biliary drainage when oral ciprofloxacin was used after six days of IV treatment [21]. Moreover, within the population of a randomized trial showing that a 1-week regimen is not inferior to a 2-week antibiotic treatment after source control in patients with Gram-negative bacteremia (mainly from UTI), 64% of short arm participants underwent oral step-down [24]. Oral-only ciprofloxacin regimen has been also applied to an older randomized trial for hospitalized adults with pyelonephritis or other complicated UTIs where 42% and 33%, respectively, had concomitant bacteremia, with low rates of poor clinical or microbiological response [25]. The upcoming results from the completed, randomized, non-inferiority SOAB (switch to oral antimicrobials in Gram-negative bacteremia) trial (NCT04146922) for individuals with bloodstream infection caused by *Enterobacterales* who are afebrile and hemodynamically stable after source control and have received a minimum of 3-day IV therapy will further enhance data in this topic [26]. Another randomized, non-inferiority trial (INVEST [NCT05199324]) for non-critically ill, non-severely immunocompromised participants with Gram-negative bacteremia is at the recruiting phase and will hopefully provide evidence about outcomes and cost-effectiveness of early (within 72 h) oral switch to ciprofloxacin or trimethoprim/sulfamethoxazole (TMP/SMX) [27].

Oral antimicrobials have been evaluated in pediatric populations against bacteremia caused by *Salmonella* spp., too. Data derived from a study in Nigeria indicate that oral ciprofloxacin for *Salmonella* bacteremia seems to be a common practice [28]. Patients receiving ciprofloxacin may have lower rates of microbiological failure and relapse compared to cefixime [29]. However, in a randomized trial from Pakistan, cefixime was as effective as ceftriaxone for bacteremic typhoid fever; more than 8-day treatment may not provide any additional benefit [30,31].

##### *Enterobacterales* Bacteremia: Oral Agent Selection

Oral switch can be performed as early as three days after IV treatment initiation, once the patient is afebrile for 1–2 days and clinical and laboratory improvement has been observed [13,17,21,23]. Concerning the choice of oral agent, most studies have used high-bioavailability drugs such as levofloxacin or moderate-bioavailability drugs like ciprofloxacin and TMP/SMX [13,21]. When the aforementioned antibiotics were the antibiotics of choice for oral step-down in an observational study of adults with bacteremic UTI performed at 24 US hospitals, no difference in odds of recurrence between patients who received 1- or 2-week regimens was observed [18]. In a meta-analysis, oral or IV fluoroquinolones were non-inferior to other antibiotics when used as definitive therapy even for bacteremia from extended-spectrum beta-lactamase (ESBL)-producing strains, if quinolone-susceptible; similar outcomes for fluoroquinolones or TMP/SMX versus carbapenems were observed against ESBL or AmpC producers [32,33]. These favorable results likely explain the higher incidence of OPAT reported in fluoroquinolone-resistant Gram-negative bacteremia [34].

Nevertheless, in addition to their high utility, fluoroquinolones can have, like any drug, toxicity issues, too. Out of all antibiotic classes, fluoroquinolones have the highest hospitalization rates due to adverse effects according to the 2013–14 US registry which reported emergency department examinations for a drug-induced etiology [35]. Of the cases, 14.5% that had an emergency visit in this study due to fluoroquinolone use needed hospital admission; the main adverse events consisted of allergic reactions, gastrointestinal symptoms (abdominal pain, diarrhea, bloating, vomiting), neuropsychiatric manifestations (seizures, lethargy, confusion, dizziness), musculoskeletal complaints such as tendinitis, and secondary infections, primarily *Cl. difficile* colitis and mucosal candidiasis [35]. Fluoroquinolones should therefore be administered with great caution and close follow-up to individuals with a history of seizures, psychiatric disease, dementia, as well as QTc prolongation.

There is also evidence, however, that if beta-lactams are appropriately dosed are equally effective, provided that are able to achieve adequate serum levels. When beta-lactam antibiotics compared with fluoroquinolones or TMP/SMX in an observational study among 4089 adults with UTI plus bacteremia, non-significant differences in UTI recurrence, 30- and 90-day bacteremia recurrence and all-cause mortality were found when individuals had previously received 4–5 days of effective IV antimicrobial therapy; treatment failure was comparable for patients who received beta-lactams and fluoroquinolones in other retrospective analyses from the US and Canada, too [19,36,37]. Similar results were reported by Mercuro et al., who also included several patients with intra-abdominal source of bacteremia [23]. In addition, patients in oral beta-lactam arm experienced significantly fewer adverse events [23]. The aforementioned study showed that a more than 10-day treatment did not have more clinical or microbiological success or less 30-day readmission rates, even for oral beta-lactams [23]. On the other hand, beta-lactam step-down therapy was inferior to fluoroquinolones or TMP/SMX in other retrospective studies for *Enterobacterales* bacteremia [22,38,39]. Oral beta-lactam administration was associated with increased risk of 90-day mortality and recurrence, mainly due to cefdinir use in some patients, which is an antibiotic not reaching adequate serum concentrations, in one study and due to low oral beta-lactam dosages in others [22,38,39,40].

##### Oral Cephalosporins for *Enterobacterales* Bacteremia: PK/PD Data

Current susceptibility breakpoints defined by European Committee on Antimicrobial Susceptibility Testing (EUCAST) and Clinical and Laboratory Standards Institute (CLSI) for oral cephalosporins cannot be implemented in bacteremia cases; for EUCAST, in particular, these breakpoints can be applied only for uncomplicated UTI. Pharmacokinetic/pharmacodynamic (PK/PD) studies showed with Monte Carlo simulations, considering the bactericidal target %*f*T > minimum inhibitory concentration (MIC) > 60–70%, that probability of target attainment (PTA) > 90% in serum could not be achieved with regular cefuroxime dosing (500 mg bid); the same results were reported with cefditoren and confirmed in another study, even when the target was %*f*T > MIC > 40% [40,41,42]. Considering these studies, cefaclor and cefprozil should not be choices for oral treatment of bacteremia, too [40,42]. Even when high-dose cefuroxime (500 mg tid) was simulated, PTA was >90% only for MIC ≤ 0.5 mg/L, thus far below the breakpoint [41]. On the other hand, cefixime reached PK/PD target for MIC ≤ 0.5 mg/L, but only when prescribed in high doses (400 mg bid); similar pharmacological targets seem to be achievable using cefpodoxime in the same dose [40,41]. Regarding cefalexin, high doses of 1 g qid and 500 mg qid can reach PTA > 90% for MIC ≤ 4 and ≤2 mg/L, respectively, if the target is %*f*T > MIC > 40%; similar findings have been also previously published [40,42]. Other study has reported PTA > 90% (with a target of %*f*T > MIC > 70%) if MIC is ≤1.5 and ≤3 mg/L for 1 g tid and 1 g qid regimens, respectively (see also Table 1) [43].

##### *Pseudomonas* Bacteremia: Clinical Efficacy Data

Ciprofloxacin and levofloxacin are to date the only studied and commercially available oral options against systemic infections caused by *Pseudomonas* spp. A retrospective study with *Pseudomonas* bacteremia cases revealed that 28-day mortality rate stratified by APACHE II and Pitt bacteremia score was not statistically different between patients who were treated with fluoroquinolone or antipseudomonal beta-lactam [50]. Notably, 44.4% of patients at the fluoroquinolone group continued the drug orally after a median of six days IV [50]. In addition, retrospective data for *Pseudomonas* bacteremia derived from a systematic review and meta-analysis did not show any worse outcomes in subjects who received fluoroquinolones, even though oral switch was applied in a substantial percentage of cases [51].

##### Oral Ciprofloxacin for Gram-Negative Bacteremia—PK/PD and TDM Data

Oral ciprofloxacin may have less bioavailability compared to newer fluoroquinolones but is still a drug of choice from this category against Gram-negative bacteria, especially in endemic tuberculosis areas where levofloxacin and moxifloxacin are reserved for these patients. Retrospective studies have shown its potency and safety against Gram-negative bacteremia regardless of the causative microorganism [52,53]. When ciprofloxacin is used for bacteremia, the efficacy target should be area under the curve (AUC)_0–24_/MIC ≥ 125, because probability for clinical and microbiological cure rises substantially above this limit [54,55]. PK/PD studies suggest high PTA for 500 mg bid orally if MIC ≤ 0.125 mg/L; 750 mg bid is expected to be adequate for MIC ≤ 0.25 mg/L, as is the dose of 1200 mg/day IV [10]. These findings are corroborated by therapeutic drug monitoring (TDM) data. When 500 mg bid regimen was prescribed against strains with an MIC of 0.25 mg/L, PTA was only 41%, which rose to 72% after 1–2 days of treatment [56]. For the EUCAST breakpoint of 0.5 mg/L for *Enterobacterales* (except *Salmonella*) and *Pseudomonas*, none of these regimens can reach PK/PD targets [10,56]. Only very high dosages (1 g bid) that are not suggested by European Medicines Agency could be sufficient [56,57]. Guideline-indicated dose reduction in patients with impaired renal function is expected to further reduce PTA because reduced renal clearance of ciprofloxacin in this context is compensated, to a great extent, by fecal elimination (see also Table 2) [56,58]. 

#### 3.1.2. Gram-Positive Bacteremia

##### *S. aureus* Bacteremia: Clinical Efficacy Data

Oral treatment for staphylococcal bacteremia is not a novel concept. Carney et al. reported a series of 18 oncology patients with staphylococcal bacteremia who were switched to oral dicloxacillin or cefalexin 1 g qid after a mean of 9-day IV therapy from as early as the 1980s, resulting in clinical and bacteriological cure in all cases except 1 [59]. Several retrospective studies have been published data about safety and efficacy of oral step-down in patients with bacteremia from *S. aureus*. Diego-Yagüe et al. found no difference in 90-day microbiological failure, relapse, and mortality when patients with uncomplicated bacteremia who had already received IV treatment for a median of one week underwent oral de-escalation compared with a cohort that received IV-only therapy; approximately one-third of the subjects received oral fluoroquinolones and another half of them oral beta-lactams [60]. Oral beta-lactams (mainly flucloxacillin 1 g tid) were also predominantly prescribed to 81 low-risk, mostly CRBSI, bloodstream infections in a retrospective study from New Zealand, when 5 days of IV treatment had preceded [61]. This approach resulted in low 90-day recurrence and mortality (4% and 2%, respectively) [61]. Another single-center study including 70 persons with variable sources of bacteremia (mainly ABSSSI) who underwent oral de-escalation reported a 7.1% 90-day clinical failure rate; 90-day readmission rate, however, was 32.9% [62]. The most commonly prescribed oral antibiotics were linezolid, TMP/SMX and clindamycin after a median of eight days [62]. 

Similar results are available even for complicated bacteremia. Pérez-Rodríguez et al. reported no differences in clinical cure, death or 90-day recurrence in an observational study that almost half of the patients had a bone infection as the source of their bacteremia, whereas hospital stay was statistically shorter in the oral step-down group (36 vs. 18 days) [63]. Oral monotherapy was administered; two-thirds of the subjects received TMP/SMX [63]. Unlike other sources of bacteremia, de-escalation to oral antimicrobials should not be applied before the first week of treatment in patients with bone and joint-originated infection based on the design of OVIVA trial [64]. In addition, a retrospective cohort analysis including *S. aureus* infective endocarditis or bacteremia with epidural abscess, vertebral osteomyelitis or septic arthritis in people who inject drugs showed that microbiological failure did not differ between patients who were discharged with oral antibiotics (either monotherapy or antibiotic combination) after a 10-day IV regimen and patients who received only parenteral antibiotics [65]. 

Regarding non-observational data, a meta-analysis of older randomized trials for linezolid in *S. aureus* ABSSSI and pneumonia revealed that patients with concomitant bacteremia had received oral linezolid after 8.6 days of IV treatment [66]. In addition, a fluoroquinolone (fleroxacin in particular)–rifampicin combination has been used orally for the whole course of treatment in *S. aureus* bacteremia versus parenteral flucloxacillin or vancomycin in a randomized trial that showed no significant difference in cure rate [67]. However, patients from the IV arm had an 11-day longer median hospital stay [67]. TMP/SMX, even in a dose of 320 mg/1600 mg bid, should not be used as monotherapy during the initial, IV part of treatment for bacteremia patients because it failed to meet non-inferiority criteria versus vancomycin, thus increasing treatment failure risk [68,69]. However, it can be used as oral step-down therapy according to all the aforementioned retrospective data, UK guidelines, as well as the results of the long awaited SABATO trial, where oral TMP/SMX 160/800 mg bid was used in 58.3% of the cases; another 32.4% of the participants received oral clindamycin 600 mg tid [68,70]. In this study, patients with low-risk *S. aureus* bacteremia were randomized to either an oral antimicrobial or to continue with IV therapy after 5–7 days of parenteral antimicrobial treatment; non-inferiority criteria for the composite primary endpoint (90-day relapse, evolution of deep-seated infection or mortality attributable to primary infection) were met [70]. These data enriched the available evidence about oral treatment in bacteremia for clinically improved patients without prolonged bacteremia, concomitant pneumonia or other deep-seated infectious focus; subjects in the oral switch group hospitalized for 3.1 days less, too [70].

##### *S. aureus* Bacteremia: Duration of Treatment

Less than 2-week regimens should generally be avoided given the findings of Chong et al., where no relapses of uncomplicated *S. aureus* bacteremia occurred in patients who were treated for ≥14 days [71]. Duplex ultrasonography may be helpful in order to distinguish cases of septic thrombophlebitis in apparently uncomplicated cases of staphylococcal CRBSI that could lead to the appropriate extension of treatment duration and consequently less relapse rates [72].

##### Linezolid for *S. aureus* Bacteremia

Linezolid is suggested as the first-choice alternative drug for methicillin-resistant *S. aureus* (ΜRSA) bacteremia by the UK guidelines when vancomycin is contraindicated [68]. This recommendation may be easily extrapolated to oral linezolid, given the fact that this drug has excellent oral bioavailability. It should be noted here that an older randomized trial has showed that linezolid was non-inferior to vancomycin or oxacillin in patients with Gram-positive, mostly staphylococcal, CRBSI in terms of clinical or microbiological failure and mortality [73]. Good results (17.1% 90-day infection-related readmission, 4% 90-day mortality) have been also published from 54 patients who received oral linezolid after a median of 5-day IV treatment; these subjects experienced significantly less line-associated and total adverse events requiring readmission compared with vancomycin or daptomycin OPAT [74]. Another study for oral linezolid in this setting was a prospective cohort study from Spain with mostly uncomplicated catheter and ABSSSI-originated bacteremia [75]. Oral switch after a median of one week resulted in 30-day mortality and 90-day relapse rates comparable to conventional parenteral regimens; patients from the IV-only group, however, had a longer hospital stay (19 vs. 8 days) [75].

##### Clindamycin for *S. aureus* Bacteremia

Clindamycin, on the other hand, had been prescribed as oral monotherapy, mostly when ABSSSI was the source of bacteremia, after a median of six days of IV antimicrobials in a retrospective study from Australia [76]. It is notable that, when the source of bacteremia is osteomyelitis, a classic oral dosage of 600 mg tid is probably suboptimal in patients weighing >75 kg according to a PK/PD study using data from clindamycin TDM in 50 patients with bone infection; 900 mg tid is needed in such cases (see also Table 2) [77]. The reasons for this are the augmented clindamycin clearance when body weight increases, despite an oral bioavailability of 87.6%, and clindamycin bone penetration rate of approximately 30% [77,78]. Oral clindamycin should not be co-administered with rifampicin unless clindamycin TDM is available because in a study for osteoarticular infections, clindamycin serum concentrations (trough and peak) were systematically below the recommended therapeutic targets when combined with rifampicin [79,80]. In contrast, this was not the case neither for IV clindamycin in continuous infusion when co-administered with rifampicin nor for clindamycin–levofloxacin combination [79,80]. In particular, data from an observational PK study comparing the effect of rifampicin on IV and oral clindamycin metabolism revealed that route of administration plays a major role; when oral clindamycin was given, AUC_0–8h_ decreased 12 times (3.1 versus 37.7 mg·h/L) compared with the same continuous IV dose [80]. Because rifampicin has only a small influence on clindamycin half-life, this effect is probably due to increased hepatic first-pass metabolism of clindamycin in the case of oral intake, thus reducing clindamycin oral bioavailability from 59.7% to 15.1% in this study [80].

##### Fluoroquinolones for *S. aureus* Bacteremia

Fluoroquinolone monotherapy against *S. aureus* is generally not preferred in most guidelines due to on-treatment resistance issues irrespective of infection type. However, not all antibiotics of this class are the same. Moxifloxacin seems to be the most potent “anti-staphylococcal fluoroquinolone”. An older PD model has showed that the equivalent daily dose of 400 mg of moxifloxacin against *S. aureus* is 5000 mg of levofloxacin, well above the studied human daily doses, whereas in another model ciprofloxacin was probably suboptimal even in a dosage of 750 mg bid [81,82]. The MIC breakpoint for killing and resistance suppression for moxifloxacin 400 mg qd should be ≤0.06 mg/L based on a pharmacometric PK/PD model derived from plasma and target site TDM, far below the established breakpoint of 0.25 mg/L; microbial subpopulations with acquired resistance to moxifloxacin could emerge if used as monotherapy above this threshold [83]. High-dose moxifloxacin (800 mg/day) can increase this breakpoint to ≤0.125 mg/L, which is a dose that has been successfully used for tuberculosis; further data are needed before applying such strategy in everyday routine (see also Table 2) [83,84].

Moxifloxacin has also been effectively used in other biofilm-associated infections caused by *S. aureus* like orthopedic implant-related infections, either as monotherapy or in combination with rifampicin, even though studies on tuberculosis have shown that rifampicin lowers moxifloxacin serum levels by approximately 30% [85,86,87,88]. Relapses in such infections were not attributed to on-treatment moxifloxacin resistance, as opposed to when ciprofloxacin had been previously used as monotherapy [85,86,89].

##### Rifampicin-Containing Regimens for *S. aureus* Bacteremia

Adjunctive rifampicin seems to be a reasonable approach in patients with bacteremia from rifampicin-susceptible strains who have a (yet uninfected) foreign body. Two post hoc analyses of INSTINCT cohort study showed that this subgroup had a significant reduction in bacteremia-related late complications and 90- and 180-day mortality when received a biofilm-acting agent like rifampicin, fluoroquinolone or fosfomycin as second antibiotic [90,91]. This result is strengthened by the observation that 68% of bacteremia-related late complications were foreign body-associated [91]. Notably, moxifloxacin should not be combined with doxycycline against *S. aureus* because available data indicate antagonism between these drugs [92]. 

##### Streptococcal Bacteremia: Clinical Efficacy Data

Multiple retrospective studies have been published on streptococcal bacteremia regarding oral switch, too. More than two decades ago, a case series with 18 individuals hospitalized for bacteremic *Streptococcus pneumoniae* pneumonia with intact gastrointestinal absorption who underwent oral switch within the first week of hospitalization was published [93]. All patients had been improved clinically before switch and no one experienced a failure [93]. Moreover, data from a randomized trial comparing two amoxicillin/clavulanate oral formulations for community-acquired pneumonia show that 37 of these participants had bacteremic pneumonia from *S. pneumoniae* and received only oral antibiotics for the whole course of treatment; no worse outcomes were reported for this subgroup [94]. 

In a subsequent cohort of 244 patients with streptococcal bacteremia without endocarditis, concomitant bone and joint or central nervous system infection who had initial Pitt bacteremia scores ≤ 3 and had already improved from IV treatment, 40% underwent oral de-escalation; no difference in 30-day recurrence, readmission, or mortality rate was observed [95]. The most frequent sources of bacteremia were pneumonia and ABSSSI [95]. Regardless of source or the causative species, oral step-down was performed after four days of IV therapy and total length of treatment was two weeks; this early switch reduced the duration of hospital stay by five days [95]. Oral agents of choice were fluoroquinolones and secondarily amoxicillin, clindamycin, and TMP/SMX [95]. Another two recently published cohorts with similar size, sources of bacteremia and timing of oral antibiotic initiation did not detect any significant differences in antibiotic-related adverse events and 90-day hospital readmission, treatment failure, or mortality between oral step-down and IV-only groups [96,97]. Reports with smaller number of patients almost reproduce the aforementioned results. Ramos-Otero et al. found that patients who received an oral regimen had significantly earlier hospital discharge without increased risk of recurrence or death in the same setting, irrespective of whether they received IV antibiotics for three days or more [98]. 

In addition, a multicenter study comparing oral step-down with fluoroquinolones or beta-lactams in a population consisting mostly of patients with pneumonia or ABSSSI with positive blood cultures was conducted [99]. Beta-lactams were non-inferior to fluoroquinolones for the composite outcome of 90-day recurrence, readmission, or death; in both arms one of these complications occurred at <10% of the cases [99]. Oral step-down before day 3 was identified as a risk factor for clinical failure, although the median timing was 5.67 days; total treatment duration was again two weeks [99]. On the other hand, regarding beta-hemolytic *Streptococci* in patients with ABSSSI-derived bacteremia, a study using propensity score-matched analysis could not confirm non-inferiority of oral switch to beta-lactams compared with IV antibiotics for the whole course of treatment, likely due to underdosing of oral antibiotics and the use of cefdinir, which has low bioavailability and consequently it is not an appropriate choice for bacteremia; treatment failure in both subgroups (low oral dosage and cefdinir use) was above 40% [100].

##### Enterococcal Bacteremia: Clinical Efficacy Data

Regarding enterococcal bacteremia, ampicillin or amoxicillin should be the backbone of antimicrobial therapy if the causative strain is susceptible, because data from a retrospective cohort study conducted in Australia revealed that glycopeptide administration instead of beta-lactams was an independent risk factor for 30-day mortality [101]. Notably, these results favored beta-lactams despite including in the cohort patients with endocarditis who were almost exclusively in the beta-lactam group; individuals in vancomycin arm were younger, too [101]. These findings were in alignment with a subsequent retrospective study where 30-day mortality rate was higher in the vancomycin treatment group, even though a percentage of patients in the anti-enterococcal beta-lactam group were switched to oral amoxicillin at some point during treatment [102]. It is unknown, however, whether these results suggest that ampicillin is truly superior compared with vancomycin or this is a consequence of vancomycin underdosing, which is frequent in the absence of TDM if a conventional 1 g bid dose is applied [103]. 

For infections caused by ampicillin-resistant strains, glycopeptides remain the first choice during initial management due to efficacy and lower cost. Linezolid, however, could also be administered, either in patients who experience adverse events from glycopeptides or as an oral step-down approach. Data from the previously mentioned randomized trial published by Wilcox et al., which included participants with enterococcal CRBSI, indicate that linezolid was non-inferior to vancomycin [73]. Moreover, a small retrospective study from Spain found no significant difference in clinical or microbiological cure and 30-day mortality among patients with bacteremia caused by *E. faecium* treated with linezolid or glycopeptides [104].

On the other hand, the optimal initial antimicrobial agent against vancomycin-resistant *Enterococci* (VRE) remains unknown due to the lack of high-quality data. A retrospective, propensity score-matched cohort study with 2630 patients who received linezolid or daptomycin for VRE bacteremia or endocarditis revealed that daptomycin group had lower mortality only for endocarditis; patients in daptomycin arm experienced fewer adverse effects, too [105]. However, it should be noted that daptomycin was prescribed in a dose of approximately 6 mg/kg (not a high dose) [105]. Furthermore, daptomycin MICs were not reported, which is the main factor that guides the optimal dose. EUCAST guidance and other publications suggest that if daptomycin MIC is ≤1 mg/L, a 6 mg/kg dose is efficacious [106]. For an MIC above 1 mg/L, however, higher doses and probably combination with a beta-lactam are needed [106,107]. In a meta-analysis concerning only high-dose daptomycin versus linezolid, again no difference in mortality was observed, although patients who received daptomycin had a higher proportion of organ dysfunction and hematologic malignancy; thrombocytopenia was significantly more frequent in linezolid group [108]. Regardless, oral de-escalation with linezolid seems like it could be implemented in any case.

##### Linezolid for Gram-Positive Bacteremia: PK/PD and TDM Data

Linezolid was prescribed as a fixed regimen of 600 mg bid in all the aforementioned studies, regardless of renal and hepatic function, as the drug summary of product characteristics indicates. These recommendations, however, are based on an older PK study where participants received only one 600 mg dose of linezolid orally [109]. The truth is that linezolid is primary metabolized in the liver and a 30% of the dose undergoes renal elimination [110]. This one-size fits all approach can lead to high interindividual variability observed by TDM, resulting in a proportion of patients treated with suboptimal doses and another percentage that is exposed to a high, myelotoxic dose. For that reason, linezolid TDM is suggested in critically ill patients and a Chinese expert consensus statement for linezolid dose optimization has been published in 2022 [111,112]. Data from a meta-analysis including 3580 patients who were prescribed linezolid revealed that eGFR ≤ 50 mL/min raised the risk for thrombocytopenia more than two-fold, and the risk become even higher as kidney function worsened or hemodialysis was needed; renal impairment was a risk factor for linezolid-induced anemia, too [113,114]. In addition, a smaller retrospective study has showed that as eGFR declines, the incidence of linezolid discontinuation due to thrombocytopenia increases and, in many cases, it may start within the first week of treatment [115]. This risk can be lowered if a dose of 300 mg bid is administered when eGFR ≤ 60 mL/min, as PTA (trough linezolid levels > 2 mg/L) was not different for this dosage compared with 600 mg bid in this subgroup according to retrospective TDM data, whereas the probability of potentially toxic trough levels (>8 mg/L) was almost three times lower (Table 2) [116]. Similar dose reductions are suggested for severe hepatic impairment (Table 2) [112,117]. On the other hand, PK/PD studies have indicated that PTA is very low when MIC is at the EUCAST breakpoint of 4 mg/L and therefore linezolid should be avoided, while PTA > 85% was achieved only through continuous infusion in critically ill patients if MIC = 2 mg/L [118,119]. Appropriate dose modification should be applied for patients with obesity, too (see Table 2) [112,119,120].

**Table 2:** Oral antibiotic dosages for bacteremia and infective endocarditis/non-beta-lactams.

Oral Antibiotic	Microorganism	MIC (mg/L)	Dosage	Dose Adjustment for Special Populations	References
Ciprofloxacin	*Enterobacterales*; *Pseudomonas*	≤0.125	500 mg bid	eGFR ≤ 30 mL/min: 25% dose reduction Obesity: no dose modification	[10,56,58,121]
		0.25	750 mg bid		
		>0.25	Avoid		
Levofloxacin	*Enterobacterales*; *Pseudomonas*; *Streptococci*	≤1	Dose guided by creatinine clearance based on ideal body weight	≤30 mL/min: 500 mg qd 30–90 mL/min: 750 mg qd >90 mL/min: 500 mg bid	[122,123,124]
		>1	Avoid		
Moxifloxacin	*Enterobacterales*;	≤0.25	400 mg qd	Renal impairment or obesity: no dose modification	[83,84,125]
	*Streptococci*	>0.25	Avoid		
	*Staphylococci*	≤0.06	400 mg qd		
		0.125	Use in combination with another agent or 800 mg/day, preferably with TDM		
		>0.125	Avoid		
TMP/SMX	*Enterobacterales*; *Streptococci*; *Staphylococci*	≤1	10 mg/kg/day (TMP component)	eGFR ≤ 30 mL/min: 50% dose reduction	[126,127]
		(as TMP concentration [=20 mg/L TMP/SXT])	divided in 2–3 doses		
		>1	Avoid		
Clindamycin	*Staphylococci*; *Streptococci*	≤0.25	600 mg tid	Bone source of bacteremia and either body weight >75 kg or MIC = 0.25 mg/L: 900 mg tid	[70,76,77,79,128,129]
		>0.25	Avoid		
Linezolid	*Staphylococci*; *Streptococci*; *Enterococci*	<2	600 mg bid	eGFR ≤ 60 mL/min, Child-Pugh C cirrhosis or INR > 2 due to hepatic impairment: 300 mg bid Dialysis-dependent patients: 300 mg bid or 600 mg qd eGFR ≥ 120 mL/min: avoid Obesity plus eGFR ≤ 60 mL/min: 600 mg bid Obesity plus eGFR ≥ 60 mL/min: 450 mg tid If body weight is >140 kg, use only if MIC ≤ 1 mg/L	[112,113,116,117,118,119,120,130,131,132,133,134,135]
		2	Use in combination if possible or use TDM		
		>2	Avoid		
Rifampicin	*Staphylococci*; *Streptococci*; *Enterococci* (adjunct treatment)	≤0.06	300 mg tid	None	[136,137]
Metronidazole	Anaerobes	≤4	Infections outside central nervous system: 500 mg bid	Obesity: 500 mg tid	[138,139,140]

List of abbreviations: MIC, minimum inhibitory concentration; eGFR, estimated glomerular filtration rate; qd, once a day; bid, two times a day; tid, three times a day; INR, international normalized ratio; TDM, therapeutic drug monitoring; TMP, trimethoprim; SXT, sulfamethoxazole.

##### Oral Amoxicillin for Bacteremia: PK/PD Data

Although oral amoxicillin is widely used against enterococcal and streptococcal bacteremia, as well as in amoxicillin-sensitive *Enterobacterales* bacteremia, oral dosing regimens are nowhere near IV dosages of 100–200 mg/kg/day [137]. The reason for this discrepancy is that such high doses are impossible to be absorbed from human gut due to the presence of a saturable, probably capacity-limited and carrier-mediated mechanism of transport from human intestine to circulation [141]. A pharmacological study in healthy volunteers has revealed that amoxicillin oral bioavailability is dose-dependent; from almost 100% for a 375 mg dose to 55% for a 3000 mg dose, which in addition caused more gastrointestinal adverse effects [141]. In alignment with these results, when amoxicillin was administered orally to individuals with an ileostomy, a 8% of amoxicillin was recovered from ileal fluid for a dose of 375 mg while for a 6000 mg dose a 77% recovery was observed, with the respective variations at the fraction excreted in urine (70% recovery at the lowest dose to 23% at the highest dose); these results have been reproduced in another study, too [142,143].

The aforementioned data play a pivotal role in the selection of patients with bacteremia or endocarditis that could be de-escalated to oral amoxicillin. A PK/PD study for pyelonephritis (where tissue and not urine antibiotic concentrations are relevant to cure) has showed that even for the modest target of 32.5%*f*T > MIC, PTA is <90% for a 1000 mg tid regimen when MIC is > 2 mg/L, well below the established breakpoint of 8 mg/L for IV administration [47,144]. A recent publication claims that maybe lower doses (750 mg tid) are sufficient for 50%*f*T > MIC if MIC ≤ 2 mg/L (see also Table 1) [10]. The same model calculated that for an MIC of 8 mg/L, an oral dose of 2500 mg tid would be necessary, which is a regimen that, while it has not been tested on a large scale, could have many tolerance issues [47]. Further evidence about the efficacy and tolerability of these higher amoxicillin doses will be provided by the awaited RODEO 2 trial (NCT02701595) for endocarditis, where 2000 mg tid oral regimens will be opted [145]. This rationale extends to amoxicillin/clavulanate, too, because PK/PD data from healthy volunteers revealed that 40%*f*T > MIC and >97.5% PTA is achieved only if MIC ≤ 1 mg/L for 500/125 mg qid and 1000/125 mg tid doses [48]. These findings corroborated by the PK/PD study from Yamada et al.; 1000/125 mg tid dose had PTA > 90% for 32.5%*f*T > MIC only if MIC was ≤2 mg/L, and 500/125 mg tid regimen had similar effect only for strains with an MIC at or below 1 mg/L [42].

### 3.2. Infective Endocarditis

#### 3.2.1. Clinical Efficacy Data and Relevant Guidelines

Although parenteral-only antimicrobial therapy for endocarditis appeared as a “rule of medicine” for decades, a series with 35 cases of *S. aureus* endocarditis who received an oral antistaphylococcal penicillin or clindamycin after a mean of 16-day IV therapy has been published in 1980 [146]. In addition, a randomized trial concerning oral treatment for right-sided staphylococcal endocarditis in people who inject drugs has been finished more than 27 years ago [147]. In this study, oral ciprofloxacin-rifampicin combination treatment was used after five days of IV antibiotics, with good results. However, the results may overestimate the efficacy of this oral regimen in endocarditis because participants who had two or more positive sets of blood cultures with no other apparent source of infection were enrolled as endocarditis cases [147]. Nowadays, VIRSTA and DENOVA scores can be applied to rule out endocarditis without cardiac ultrasound in some patients with bacteremia from *Staphylococcus aureus* and *Enterococcus faecalis*, respectively [148,149,150]. 

Both the 2015 American and European guidelines for endocarditis had already adopted oral treatment for infections caused by typical bacteria, either as a drug-of-choice or as an alternative. In particular, linezolid (oral or IV) is one of the first-line regimens for endocarditis caused by *Enterococci* that are resistant to ampicillin and vancomycin [136,151]. The efficacy of oral linezolid in this setting had already been evaluated in a small number of cases since its first years on the market [152,153]. However, linezolid treatment for enterococcal endocarditis when the pathogen is resistant to vancomycin was associated with higher mortality and adverse event rate compared with daptomycin in a retrospective cohort; therefore, it should be reserved as an oral step-down choice after the initial IV treatment with a daptomycin-containing regimen, provided that MIC for daptomycin is not >4 mg/L [105,107]. In addition, rifampicin (oral or IV) is included in the proposed regimen for staphylococcal prosthetic valve endocarditis at a dose of 300 mg tid [136,151].

Moreover, both American and European guidelines include IV or oral ciprofloxacin as an alternative treatment for HACEK endocarditis [136,151]. European guidelines also suggest the 1-week IV combination of TMP/SMX 4800/960 mg/day and clindamycin 600 mg tid, followed by oral TMP/SMX monotherapy as another option against staphylococcal endocarditis [151]. This recommendation had been based on a preliminary report of the study published by Tissot-Dupont et al. in 2019 [128]. In this study, all patients received this regimen except those who had positive blood cultures after the second day of treatment or in the cases where a cardiac abscess was present; in these two scenarios, IV rifampicin (1800 mg/day) and gentamicin (180 mg/day) were added for the first week [128]. The outcomes of the participants in this protocol were compared with an older control group of patients treated in the same center for staphylococcal endocarditis with conventional IV regimens. Although the mean age was higher in the oral group, no statistical difference in 90-day mortality or relapse was found; mortality was not significantly different even after 1-year follow-up [128]. To our opinion, further trials should be performed to assess the efficacy of clindamycin plus TMP/SMX for the whole course of treatment for endocarditis.

The publication, however, that brought the biggest changes in this setting at the recent 2023 European guidelines for endocarditis was POET trial and its sub-studies. This randomized, non-inferiority trial compared oral step-down with IV-only treatment in 400 participants with staphylococcal, enterococcal, or streptococcal left-sided endocarditis who had already received IV antibiotics for at least ten days (median 17 days) and for at least seven days after valve surgery, showed clinical and laboratory improvement, and had no indication of a local complication requiring surgery according to transesophageal echocardiography performed before randomization [44]. Notably, enrollment included participants with prosthetic valve endocarditis or a permanent pacemaker [44]. Oral de-escalation strategy, which consisted of two antibiotics with different mechanisms of action, was not inferior according to the 6-month composite outcome of mortality, unplanned cardiac surgery, embolic events, or relapse; these results were consistent during an extended follow-up after three and five years, too [44,154,155]. Moreover, the oral group had a significantly shorter length of stay in the hospital [44]. Before POET, a cohort study including 214 patients with endocarditis who underwent oral step-down had similar results; no increased risk of mortality or relapse was observed [156]. Although de-escalation was applied later in-course of treatment in this cohort (mean after 14 days for *Streptococci* and 28 days for *S. aureus* and *Enterococci*) it still reduced IV-treatment days substantially, given the fact that 23% of the cases had prosthetic valve endocarditis [156]. Patients were eligible for oral treatment only if they were afebrile, laboratory and imaging abnormalities were improving, and blood cultures had been sterilized [156]. Oral combination treatment was prescribed only against *Staphylococci* (mostly two of a fluoroquinolone, clindamycin, and rifampicin or combination of amoxicillin with one of them), whereas a strategy of amoxicillin monotherapy was almost totally adopted for *Streptococci* and *Enterococci* [156].

The more commonly used oral regimens in POET trial were rifampicin 600 mg bid plus either amoxicillin or dicloxacillin 1 g qid for *S. aureus* (all cases were methicillin-sensitive), amoxicillin as backbone therapy plus one of rifampicin, moxifloxacin and linezolid against *E. faecalis*, and for *Streptococcus* spp. endocarditis, the aforementioned anti-enterococcal regimens were mainly prescribed, as well as a linezolid–rifampicin combination [44]. All these findings led 2023 European Society of Cardiology guideline task force to adopt the rationale of POET study and to suggest oral step-down with two antibiotics from different drug classes, provided that all criteria applied in the study are fulfilled [137]. Proposed regimens from the guidelines are similar to those used in POET.

Another oral antibiotic combination that, to our perspective, appears promising against endocarditis caused by *E. faecalis* is amoxicillin plus cefditoren, an “oral extrapolation” of the IV, guideline-proposed ampicillin–ceftriaxone regimen [136,137]. Attanasio et al. published a case series of five patients with *E. faecalis* prosthetic valve endocarditis who received amoxicillin/clavulanate 1 g tid plus cefditoren 400 mg bid as an oral regimen after a median of four weeks IV; no patients required surgery [157].

#### 3.2.2. Considerations Regarding Oral Antibiotic Selection in Endocarditis

Notably, except the aforementioned combinations, guidelines also include regimens that did not tested thoroughly in the POET study like linezolid plus rifampicin for *Staphylococci* and *Enterococci* (two patients received it for this indication) and moxifloxacin plus rifampicin for *Staphylococci* and *Streptococci* (prescribed to three participants with staphylococcal endocarditis) [44,137]. Oral combination therapy with fucidic acid 750 mg bid is also included as a potential approach, although only six patients received it in POET [44,137]. 

**Figure 1:**
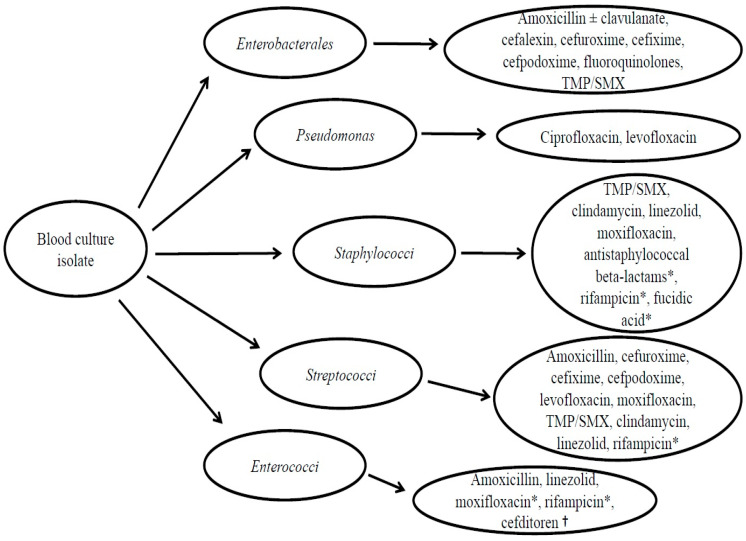
Oral antibiotic options for bacteremia and endocarditis according to causative pathogen, provided that PK/PD targets can be achieved given the MIC. * Only combined with another active antibiotic. † Only as an adjunct treatment against *E. faecalis.* List of abbreviations: TMP/SMX, trimethoprim/sulfamethoxazole; PK/PD, pharmacokinetic/pharmacodynamic; MIC, minimum inhibitory concentration.

Like previously mentioned, moxifloxacin presents antagonism with doxycycline and moxifloxacin levels are reduced when co-administered with rifampicin; dramatic reduction in oral clindamycin bioavailability has also been observed if used with rifampicin. Okazaki et al. have reported reduced linezolid serum concentration when combined with rifampicin [158]. Consequently, to our opinion, the aforementioned regimens should be avoided until further data are available. Even if linezolid–rifampicin co-administration has been effectively used for tuberculosis, that is a totally different context consisting of multidrug treatment against a microorganism that has substantially lower MIC for linezolid, so these data should not be extrapolated to endocarditis or bacteremia. Another drug class that should be used with extreme caution against *S. aureus* endocarditis is oral beta-lactams. This conclusion is derived from a PK/PD study based on TDM results from POET trial, where using a clinical breakpoint for staphylococci, the PTA for dicloxacillin was 9%–17% [159]. A schematic illustration of the current antibiotic armamentarium depending on blood culture result is provided in Figure 1.

#### 3.2.3. Probenecid as an Adjunct to Oral Beta-Lactams

A promising solution in improving the PTAs of oral beta-lactams is the addition of probenecid, a molecule that acts through competitive inhibition of organic anion transporters, which excrete beta-lactams from the kidneys. Probenecid remains a recommended adjunct against some sexually transmitted diseases like neurosyphilis [160]. Moreover, oral amoxicillin can achieve treponemicidal levels in cerebrospinal fluid only when combined with probenecid [161]. A systematic review and meta-analysis including data mainly from healthy volunteers suggested that when probenecid is added to an oral beta-lactam increases total AUC, maximum concentration and serum half-life, thus improving PTA [162]. In particular, study results from a PK/PD model that was developed with data from co-administration of 1 g cefalexin with 500 mg of probenecid in 11 healthy individuals showed that PTA of %*f*T > MIC > 70% if MIC is 8 mg/L (epidemiological cut-off value (ECOFF) for *S. aureus*) rises from <15% for cefalexin alone to almost 100% for a cefalexin 1 g qid regimen plus probenecid 500 mg bid [43]. If MIC is ≤4 mg/L, then a regimen of cefalexin 1 g tid plus probenecid seemed sufficient [43]. Administration with food and maybe even smaller doses of probenecid in cases of renal impairment can reduce probability for drug-induced nausea or other gastrointestinal adverse events [43]. In comparison, PTA > 90% was achieved if MIC was ≤1.5 and ≤3 mg/L for oral cefalexin alone when administered as 1 g tid and 1 g qid regimen, respectively [43].

#### 3.2.4. Infective Endocarditis—Upcoming Trials

There is no doubt that further randomized trials are needed for bacterial endocarditis in order to solidify the evidence about the timing of oral switch and the proper oral regimens against each microorganism. The RODEO 1 (NCT02701608) and RODEO 2 (NCT02701595) trials for left-sided endocarditis caused by *S. aureus* and *Streptococcus*/*Enterococcus* spp., respectively, are in the recruitment phase [145]. Oral treatment will be attempted in both studies after a minimum of ten days IV therapy. Oral levofloxacin–rifampicin combination will be studied in RODEO 1 and oral amoxicillin in high doses (1500 mg tid for patients ≤70 kg and 2000 mg tid for patients >70 kg) in RODEO 2 [145]. In addition, another trial (NCT04544306) examining non-inferiority of oral treatment after at least a 10-day IV regimen for endocarditis in people who inject drugs is recruiting participants [163].

#### 3.2.5. The Potential Utility of Oral Fosfomycin against Endocarditis and Bacteremia

**Figure 2:**
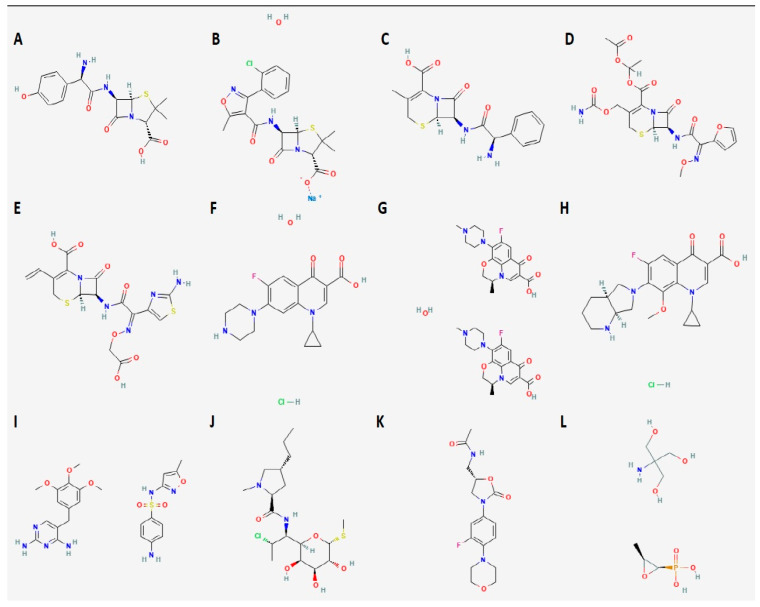
Chemical structure of certain commonly used oral antibiotics (adapted from PubChem): (**A**), amoxicillin; (**B**), cloxacillin sodium; (**C**), cefalexin; (**D**), cefuroxime axetil; (**E**), cefixime; (**F**), ciprofloxacin hydrochloride; (**G**), levofloxacin hemihydrate; (**H**), moxifloxacin hydrochloride; (**I**) sulfamethoxazole/trimethoprim; (**J**), clindamycin; (**K**) linezolid; (**L**) fosfomycin trometamol [180,181,182,183,184,185,186,187,188,189,190,191].

Fosfomycin is a hydrophilic antibiotic with small molecular mass which has negligible protein binding and consequently has good tissue distribution (see also Figure 2) [164,165]. In addition, it seems that it has some yet unclear PK/PD properties [166]. Time-dependent killing has been identified against *S. aureus* and *S. pyogenes*, whereas concentration-dependent activity has been found against *E. coli* and *Proteus* [167,168,169].

IV fosfomycin has already shown its effectiveness in the context of bacteremia and endocarditis. It was non-inferior to piperacillin/tazobactam as monotherapy in ZEUS trial which enrolled cases with complicated UTI, including patients with concomitant bacteremia [170]. Fosfomycin has also showed synergistic effects with many antimicrobials and for that reason antibiotic combinations including fosfomycin are therapeutic options for staphylococcal and enterococcal endocarditis [137]. Moreover, it can act against infections where biofilm formation is involved (e.g., prosthetic joint infections, prosthetic valve endocarditis) [90,91,137]. 

When the linezolid–fosfomycin combination was used at clinical isolates of *Enterococcus*, it prevented the emergence of resistant mutants in lower drug concentrations and was synergistic in a hollow fiber model; another in vitro study demonstrated a reduction in linezolid MIC when co-administered with fosfomycin, even though MICs for fosfomycin were relatively high (≥64 mg/L) [171,172,173]. The simulated fosfomycin regimen in this work, however, was higher than the levels achievable by oral administration [171]. In addition, an in vitro study showed that the fosfomycin–cefixime combination has synergistic killing properties against *E. coli*, even in half the MIC concentrations for each drug [174].

Oral fosfomycin trometamol exhibits a 33–44% bioavailability [166,175,176,177]. This characteristic has precluded its use for infections outside urinary tract until now. To date, clinical data from oral fosfomycin in this context do not exist. Khatri et al. reported two cases with persistent VRE bacteremia in neutropenic patients despite a multiple antibiotic treatment regimen that resolved only when oral fosfomycin in conventional doses was added [178]. According to a population PK model, oral fosfomycin in higher doses than 3 g qd should be studied as a potential therapy for the treatment of systemic infections [179]. This model indicates that an oral dose of 3 g tid can achieve the time and concentration-dependent targets for gram-positives, *E. coli* and *Proteus* if MIC ≤ 8 mg/L [179]. The simulation for an even higher oral dose (6 g TID) revealed a %*f*T > MIC of 100% for MICs ≤ 16 mg/L and a maximum concentration >4 x MIC even for MICs above 8 mg/L (e.g., 12 mg/L) [179].

## 4. Conclusions

In summary, it seems like the available data, although the majority of them are non-randomized, are too significant to ignore; they suggest that, when a patient with bacteremia has received IV antibiotics and has improved, then it is safe and effective to de-escalate—after appropriate source control—to oral antibiotics, which have been shown to achieve their PK/PD target in serum. This strategy can reduce patient discomfort, hospital-associated complications, and costs. Until now, examples where IV therapy should be extended are concomitant bone infection and endocarditis, but even these cases can be safely switched after 7 and 10 days IV, respectively, based on randomized studies. It is yet unknown what the optimal approach should be to an individual who has a community-acquired infection but does not meet criteria for hospital admission, so empiric oral antibiotic treatment is prescribed, and subsequently blood cultures turn positive. Based on the scarce aforementioned data concerning oral-only regimens against bacteremia, if the clinical condition remains good, then an outpatient therapy could be tried with close follow-up examinations, but this is definitely a matter for debate. It is a fact that there are still many unanswered questions regarding antibiotic selection, the choice of monotherapy or combination treatment, antibiotic dosage, the timing of the oral switch, and the total days of treatment that future trials could focus on and consequently lead more physicians to implement this concept into their everyday practice.

## Figures and Tables

**Table 1:** Oral antibiotic dosages for bacteremia and infective endocarditis/beta-lactams.

Oral Antibiotic	Microorganism	MIC (mg/L)	Dosage	Dose Adjustment for Special Populations	References
Amoxicillin	*Enterobacterales*; *Streptococci*; *Enterococci*	≤1 1–2 >2	1 g tid 1 g qid Avoid	eGFR ≤ 10 mL/min or dialysis-dependent patients: 1 g bid	[40,44,45,46,47]
AMX/CLAV	*Enterobacterales*	≤2 >2	1000/125 mg tid Avoid	eGFR ≤ 10 mL/min or dialysis-dependent patients: 1000/125 or 875/125 mg bid	[42,46,48]
Cefalexin	*Enterobacterales*	≤1.5 ≤3 >3	1 g tid 1 g qid Avoid	eGFR 10–30 mL/min: 1 g tid eGFR ≤ 10 mL/min or dialysis-dependent patients: 1 g bid	[43,49]
Cefuroxime axetil	*Enterobacterales*; *Streptococci*	≤0.5 >0.5	500 mg tid Avoid	None	[41]
Cefixime - Cefpodoxime	*Enterobacterales*; *Streptococci*	≤0.5 >0.5	400 mg bid Avoid	None	[40,41]

List of abbreviations: MIC, minimum inhibitory concentration; eGFR, estimated glomerular filtration rate; bid, two times a day; tid, three times a day; qid, four times a day; AMX/CLAV, amoxicillin/clavulanate.
